# FGF10 Therapeutic Administration Promotes Mobilization of Injury-Activated Alveolar Progenitors in a Mouse Fibrosis Model

**DOI:** 10.3390/cells11152396

**Published:** 2022-08-03

**Authors:** Yu-Qing Lv, Ge-Fu Cai, Ping-Ping Zeng, Qhaweni Dhlamini, Le-Fu Chen, Jun-Jie Chen, Han-Deng Lyu, Majid Mossahebi-Mohammadi, Negah Ahmadvand, Saverio Bellusci, Xiaokun Li, Chengshui Chen, Jin-San Zhang

**Affiliations:** 1 Department of Pulmonary and Critical Care Medicine, The First Affiliated Hospital of Wenzhou Medical University, Wenzhou 325000, China; lyuyuqing@wmu.edu.cn (Y.-Q.L.); chenlefu915@outlook.com (L.-F.C.); chenjunjie@wzhospital.cn (J.-J.C.); 2 International Collaborative Center on Growth Factor Research, School of Pharmaceutical Sciences, Wenzhou Medical University, Wenzhou 325035, China; zengpingping@wmu.edu.cn (P.-P.Z.); dhlaminiqhaweni@wmu.edu.cn (Q.D.); lyu.handeng@mayo.edu (H.-D.L.); majid.mossahebi@modares.ac.ir (M.M.-M.); 3 Biomedical Collaborative Innovation Center of Zhejiang Province, Institute of Life Sciences, Wenzhou University, Wenzhou 325035, China; 194511382323@stu.wzu.edu.cn; 4 Department of Pulmonary and Critical Care Medicine and Infectious Diseases, Universities of Giessen and Marburg Lung Center, Justus-Liebig University Giessen, 35392 Giessen, Germany; negah.ahmadvand@innere.med.uni-giessen.de (N.A.); saverio.bellusci@innere.med.uni-giessen.de (S.B.); 5 Department of Pulmonary and Critical Care Medicine, The Quzhou Affiliated Hospital of Wenzhou Medical University, Quzhou People’s Hospital, Quzhou 324000, China

**Keywords:** pulmonary fibrosis, bleomycin, recombinant FGF10, alveolar epithelial progenitors, AT2 cells

## Abstract

Idiopathic pulmonary fibrosis (IPF) is a devastating interstitial lung disease with dire consequences and in urgent need of improved therapies. Compelling evidence indicates that damage or dysfunction of AT2s is of central importance in the development of IPF. We recently identified a novel AT2 subpopulation characterized by low SFTPC expression but that is enriched for PD-L1 in mice. These cells represent quiescent, immature AT2 cells during normal homeostasis and expand upon pneumonectomy (PNX) and were consequently named injury-activated alveolar progenitors (IAAPs). FGF10 is shown to play critical roles in lung development, homeostasis, and injury repair demonstrated in genetically engineered mice. In an effort to bridge the gap between the promising properties of endogenous Fgf10 manipulation and therapeutic reality, we here investigated whether the administration of exogenous recombinant FGF10 protein (rFGF10) can provide preventive and/or therapeutic benefit in a mouse model of bleomycin-induced pulmonary fibrosis with a focus on its impact on IAAP dynamics. C57BL/6 mice and *Sftpc^CreERT2/+^*; *tdTomato^flox/+^* mice aged 8–10 weeks old were used in this study. To induce the bleomycin (BLM) model, mice were intratracheally (i.t.) instilled with BLM (2 μg/g body weight). BLM injury was induced after a 7-day washout period following tamoxifen induction. A single i.t. injection of rFGF10 (0.05 μg/g body weight) was given on days 0, 7, 14, and 21 after BLM injury. Then, the effects of rFGF10 on BLM-induced fibrosis in lung tissues were assessed by H&E, IHC, Masson’s trichrome staining, hydroxyproline and Western blot assays. Immunofluorescence staining and flow cytometry was used to assess the dynamic behavior of AT2 lineage-labeled *Sftpc^Pos^* (IAAPs and mature AT2) during the course of pulmonary fibrosis. We observed that, depending on the timing of administration, rFGF10 exhibited robust preventive or therapeutic efficacy toward BLM-induced fibrosis based on the evaluation of various pathological parameters. Flow cytometric analysis revealed a dynamic expansion of IAAPs for up to 4 weeks following BLM injury while the number of mature AT2s was drastically reduced. Significantly, rFGF10 administration increased both the peak ratio and the duration of IAAPs expansion relative to EpCAM^Pos^ cells. Altogether, our results suggest that the administration of rFGF10 exhibits therapeutic potential for IPF most likely by promoting IAAP proliferation and alveolar repair.

## 1. Introduction

Idiopathic pulmonary fibrosis (IPF) is the most common form of interstitial lung disease, which almost inevitably leads to respiratory failure and patient death within five years after diagnosis [1,2]. IPF-associated respiratory failure results from the aberrant deposition of extracellular matrix (ECM) and progressive loss of lung architecture and function [3]. Activated myofibroblasts (MYF) and MYF-derived ECM have traditionally been considered central in IPF pathobiology, and hence the focus of numerous studies aimed at developing targeted anti-fibrotic therapies [4]. Pirfenidone and Nintedanib, two FDA-approved anti-fibrotic IPF therapeutics, have been shown to slow the progression of the disease [5,6,7,8,9,10,11,12,13]. However, accumulating evidence suggest a pivotal role for dysfunctional alveolar epithelial cells, particularly alveolar type 2 cells (AT2s), in the pathogenesis of several parenchymal diseases, including IPF [14,15]. In this context, AT2 loss or dysfunction due to chronic or repetitive injury may lead to the development of a profibrotic phenotype [16,17]. Therefore, mitigating AT2 loss and dysfunction may prove to be beneficial in combating IPF.

AT2 cells act as prime stem cells in adult lungs for alveolar maintenance, repair, and regeneration [18,19,20]. Accumulating studies have indicated that AT2 cells are heterogenous [21] and comprise various subpopulations defined based on multiple markers, including our recently identified AT2-Sftpc^Low^ (tdTom^Low^, aka injury-activated alveolar progenitors, IAAPs) [22]. In contrast to mature AT2-Sftpc^High^ (tdTom^High^ cells, which account for the mature AT2s expressing higher levels of *Sftpc*, *Etv5*, and *Fgfr2b*), IAAPs express lower levels of AT2 differentiation markers such as *Sftpc* as well as lower levels of the FGFR2b signaling genes *Fgfr2b*, and *Etv5,* but are enriched for the immune checkpoint programmed death ligand 1 (PD-L1). IAAPs are quiescent during normal homeostasis but proliferate and are proposed to differentiate into bona fide AT2 following lung pneumonectomy (PNX). FACS-based quantification revealed that the ratio of IAAPs to total EpCAM^Pos^ cells was significantly increased upon PNX compared to sham, whereas the AT2-tdTom^High^ to total EpCAM^Pos^ ratio remained unchanged, suggesting that IAAPs are, in fact, the significant contributor to lung regeneration, rather than the previously thought mature AT2s [22].

Fibroblast growth factor 10 (FGF10) is a multifunctional growth factor that belongs to the FGF7 subfamily of the FGF family and mainly elicits biological responses through binding to and activating FGFR2b with heparan sulfate as cofactor [23,24]. In the lung, FGF10 and FGFR2b are expressed in both the mesenchymal and epithelial cell compartments, respectively, exerting a critical role for FGF10/FGFR2b in mesenchymal–epithelial crosstalk. In addition, mesenchymal FGF10 is crucial for the lineage commitment and proliferation of epithelial cells during embryonic and postnatal development and for driving epithelial cell regeneration after injury [25]. Gupte et al. reported that *Fgf10* overexpression during different stages of the bleomycin (BLM) model resulted in a significantly reduced extent of lung fibrosis, suggesting that FGF10 may be a potential candidate for treating pulmonary fibrosis [26].

Recently, we have identified the equivalent of the mouse IAAPs in the human lungs [27]. These cells were amplified in the context of end-stage IPF, concomitant with a significant decrease in the number of mature AT2s. In addition, an in vitro culture of precision cut lung slides from donor lungs demonstrated the emergence of HTII-280^Neg^ PD-L1^Pos^ cells (likely the IAAPs) and HTII-280^Pos^ PD-L1^Pos^ cells (IAAPs differentiating into AT2s) [27]. We also reported recently that in mice, IAAPs are activated upon injury, upregulate Fgfr2b expression and proliferate. This was observed following PNX to induce compensatory growth [22], as well as following *Fgfr2b* deletion in AT2 cells to robustly ablate the AT2 lineage [28]. In both cases, we observed either no change (for the PNX) or a decrease (for *Fgfr2b* deletion in AT2s) in the number of mature AT2s. 

In an effort to bridge the gap between promising properties of endogenous *Fgf10* manipulation and therapeutic reality, we here investigated whether the administration of exogenous recombinant FGF10 protein (rFGF10) can provide preventive and/or therapeutic benefit in a mouse model of bleomycin-induced pulmonary fibrosis. In addition to the histopathological aspects, we mainly focused on establishing the dynamic profile of IAAPs following BLM injury. A combination of flow cytometry and genetic lineage tracing was used to reveal the relationship between IAAPs and mature AT2s during the fibrosis development and resolution processes and the impact of rFGF10. 

## 2. Materials and Methods

### 2.1. Animals and Drug Administration

Male C57BL/6 mice and *Sftpc^CreERT2/+^*; *tdTomato^flox/+^* mice at the age of 8 to 10 weeks old were used in this study [22]. According to the “Guide for the Care and Use of Laboratory Animals” prepared by the National Academy of Sciences and published by the National Institutes of Health (NIH publication 86-23 revised 1985). Mice were housed in a temperature-controlled facility with a 12 h light/dark cycle and allowed feeding ad libitum. All animal procedures were approved by the Institutional Animal Care and Use Committee of Wenzhou Medical University.

For bleomycin (BLM) injury, adult 8- to 12-week-old mice were intratracheally (i.t.) instilled with BLM (2 μg/g body weight) or saline for the control group. BLM injury was induced after a 7-day washout period following tamoxifen induction. For tamoxifen induction, mice were administered tamoxifen in corn oil (200 μg/g) every other day for a total of 3 intraperitoneal (i.p.) injections. For recombinant FGF10 protein (rFGF10) treatment, mice were i.t. instilled with rFGF10 (0.05 μg/g body weight) on day 0, 7, 14, 21 after BLM injury as specified, and control group mice were i.t. instilled with the same volume of saline solution. 

### 2.2. Histology and Immunohistochemistry and Hydroxyproline Measurement

The lungs were embedded in paraffin wax, fixed in 10% formalin, and processed into sections. The sections were stained either with hematoxylin–eosin (H&E) or subjected to Masson’s trichrome staining. For immunohistochemistry (IHC) staining, lung sections were subjected to deparaffinization and antigen retrieval at first, then blocked by using 10% bovine serum albumin (BSA; Solarbio, Beijing, China) at room temperature (RT) for 1 h. After incubating with primary antibodies of collagen (Abcam, Cambridge, UK; 1:100) and α-smooth muscle actin (αSMA) (Beyotime, Shanghai, China; 1:100) at 4 °C overnight, appropriate secondary antibodies conjugated with HRP were added and incubated at RT. Finally, sections were visualized using a metal-enhanced DAB substrate kit (Solarbio), followed by hematoxylin counterstaining. Pulmonary fibrosis was evaluated by measuring Masson staining and the percentage-positive area was quantified using the ImageJ software. The hydroxyproline content in the mouse lungs was determined using the Hydroxyproline assay kit (Nanjing Jiancheng Bioengineering Institute, Nanjing, China; A030-2-1) following the manufacturer’s instructions.

### 2.3. Immunofluorescence

PBS perfused lung tissue was fixed in 4% PFA and incubated at 4 °C for 24 h before embedding in OCT cryostat medium (Sakura Finetek, Torrance, CA, USA) and storing at –80 °C until sectioning into 10 µm slices. The following primary antibodies were used for staining the frozen sections: Pro-SFTPC (rabbit, Abcam, 1:100), PDPN (mouse, Santa Cruz Biotechnology, Santa Cruz, CA, USA; 1:100), PD-L1(rabbit, Abcam, 1:100), Ki67 (mouse, Invitrogen, Waltham, MA, USA; 1:100), rabbit anti-FGF10 (AP14882PU-N; Acris, Rockville, MD, USA; 1:200). The sections were first washed with PBS and incubated with antigen repair solution (Beyotime) for 5 min at RT and blocked with 5% Bovine Serum Albumin (BSA) for 30 min at RT. Then the sections were incubated with the above primary antibodies diluted in 1% BSA at 4 °C overnight. The slides were washed in PBS next day and incubated with the secondary antibodies diluted in 1% BSA for 2 h at RT. Finally, the cell nuclei were counter stained with DAPI. Images were visualized and captured by using an Olympus FV3000 confocal microscope. The ImageJ program was used to determine the positive cells.

### 2.4. Western Blot

Dissected lung tissues were placed in cell lysis buffer freshly supplemented with protease inhibitor cocktail (Sigma, Alexandria, VA, USA) and phosphatase inhibitors (Roche, Nutley, NJ, USA) and homogenized for protein extraction. The protein samples were quantitated with BCA (bicinchoninic acid) protein assay (Beyotime). Protein samples (20–50 μg) were resolved on a 10% SDS–polyacrylamide gel and transferred onto PVDF membranes (Roche, 3010040001). The membranes were blocked with 5% skimmed milk in Tris-buffered saline (TBS) at RT on a shaker for 1h and then incubated with the specific primary antibodies: Collagen I (Meridian, Beijing, China; no. 1:1000), α-SMA (Abcam, no. ab9588, 1:1000), and α-tubulin (Beyotime, AF0001 1:10,000) overnight at 4 °C. After washing with TBS-T buffer, the membrane was incubated with proper HRP-conjugated goat anti-mouse or goat anti-rabbit secondary antibody (1:10,000) at RT for 1h before detection by ECL reagent (Enhanced Chemiluminescence, Amersham, UK) and image acquisition. 

### 2.5. Lung Dissociation and Preparation of Single Cells

The mice were euthanized with 4% chloral hydrate, and upon proper exposure following standard surgical procedures, the lungs were perfused with 5 mL of PBS. A 20G Angio catheter was used for i.t. instillation, via a tracheal cannula, of 1 mL dispase solution in DMEM (1 mg/mL). Then, 0.6 mL of 1% agarose was gently administered into the lungs via the catheter, and the lungs were allowed to cool down on ice for 2 min. Individual lung lobes were dissected and put in a 50 mL conical tube containing the dispase solution, incubated at RT for 45 min on a rocker at 150 rpm. Digested lungs were decanted into a 10 cm Petri dish and supplemented with complete DMEM medium containing DNase. The lung parenchyma was gently teased away from the large airways using sharp tweezers. The Petri dish was rocked at 60 rpm for another 10 min at RT. The airways were discarded by straining the lung crude single-cell prep sequentially through 70, 40, and 20 μm strainers. Cells were finally spun down at 300× *g* at 4 °C, and the corresponding pellet was resuspended in 500 μL complete DMEM.

### 2.6. Magnetic Cell Sorting (MACS) and Flow Cytometry Analysis

The MACS^®^ Separator Kit was used to deplete CD45- and CD31-positive cells from lung single cell suspensions prepared above following recommended procedures by the manufacturers. Briefly, cell suspensions were centrifuged, with the supernatant completely aspirated before adding 100 μL of CD45 and/or CD31 MicroBeads (Miltenyi Biotec, Bergisch Gladbach, Germany) per 10 million cells in MACS buffer. The cells were gently mixed and incubated for 15 min at 4 °C, then washed in 1 mL of buffer, and resuspended in 500 μL of MACS buffer before applying on the column. Next, flow-through unlabeled cells were collected and centrifuged to pellet the cells and resuspended in 500 μL of MACS buffer containing anti-EpCAM (APC-conjugated, 1:50; Biolegend, San Diego, CA, USA) for 45 min on ice in the dark. APC-conjugated rat IgG2a (Biolegend, 1:50) was used as the isotype control. Flow cytometry analysis and data acquisition were carried out using the ACEA NovoCyte flow cytometer. Data were analyzed using FlowJo software version X (FlowJo, LLC, Ashland, OR, USA). 

### 2.7. Quantification and Statistical Analysis

For quantification of immunofluorescence, cells were counted in 10 independent 20× fields per sample. For H&E staining, fibrosis was evaluated by the Ashcroft score. For Masson and IHC staining, positive areas were isolated and calculated by ImageJ. Statistical analysis and graph assembly were carried out using GraphPad Prism 8 (GraphPad Prism Software, San Diego, CA, USA). Unpaired two-tailed Student’s t-tests determined significance. Data are presented as mean ± standard error of mean (SEM). Values of *p* < 0.05 were considered significant. The number of biological samples (*n*) for each group is stated in the corresponding figure legends. *: *p* < 0.05; **: *p* < 0.01; ***: *p* < 0.001; ****: *p* < 0.0001.

## 3. Results

### 3.1. Preventative rFGF10 Delivery Decreases Fibrosis Formation

**Figure 1:**
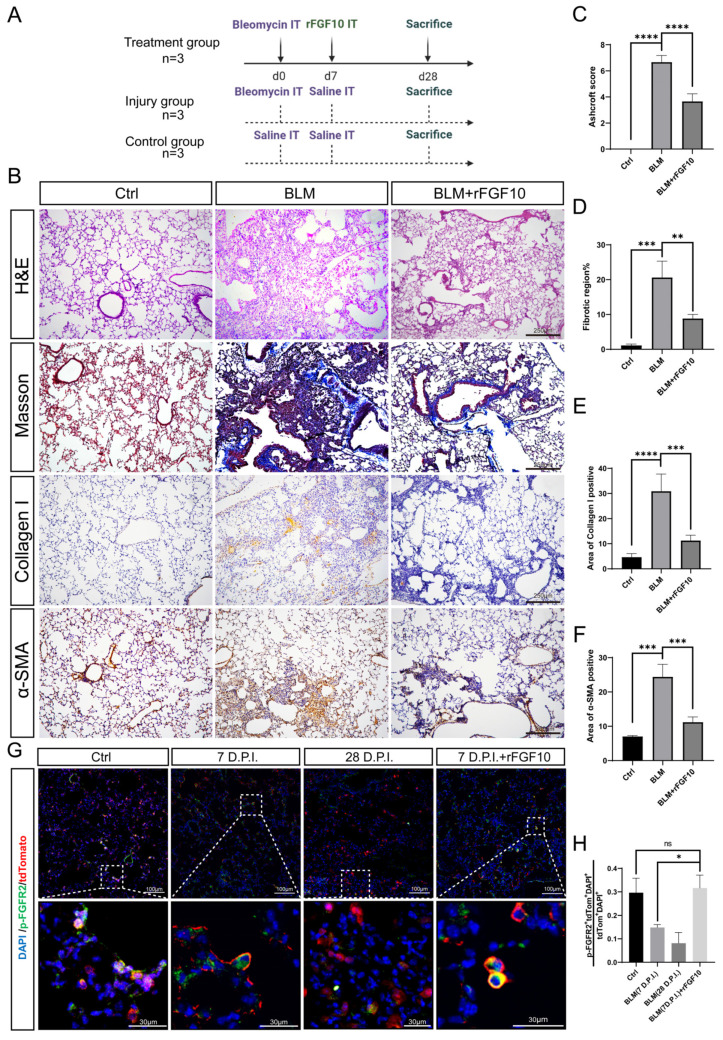
rFGF10 exhibits preventive efficacy toward BLM-induced injury. (**A**) Timelines of BLM and rFGF10 administration with saline as control; all the mice were euthanized 28 days after BLM administration (designated as Day 0). Histological analysis and quantitative fibrosis scoring of lung sections. (**B**) H&E, Masson’s trichrome and IHC staining (α-SMA, collagen) of lung tissue from control, BLM, and BLM + rFGF10 group mice. (**C**) Semi-quantitative analyses of lung tissue using the Ashcroft score (*n* = 3). Note the significantly decreased score in the BLM + rFGF10 group compared to BLM alone. (**D**) ImageJ quantification of fibrotic regions based on Masson’s trichrome staining (*n* = 3). (**E**,**F**), Quantification of collagen and α- SMA IHC staining in the lung sections of each mouse group (*n* = 3). (**G**) Immunostaining for p-FGFR2 on *Sftpc^CreERT2/+^*; *tdTomato^flox/+^* mouse lungs that received BLM at 2 months of age and were harvested at 7, 28 and 7 dpi with rFGF10 administered for 12 h, as indicated. (**H**) Quantification of immunofluorescence, showing the expression of p-FGFR2 in lineage labeled cells of the indicated groups. Data are presented as mean ± SEM. *: *p* < 0.05; **: *p*< 0.01; ***: *p* < 0.001; ****: *p* < 0.0001.

First, we examined FGF10 expression in tissue sections at three and six weeks after bleomycin injury. The immunofluorescence results showed that FGF10 expression was significantly increased in the bleomycin-injured group when compared to the control group (Figure S1). To determine the effect of exogenous FGF10 on fibrosis formation, we validated in our experimental conditions in the widely used bleomycin (BLM) model of lung fibrosis [29]. Our experimental scheme is depicted in Figure 1A. BLM-induced lung fibrosis is characterized by an acute injury, followed by localized inflammation (0–7 days), and subsequent fibrosis within four weeks [30]. We could recapitulate critical features of human IPF by i.t. instillation of BLM in mice and observed that the administration of rFGF10 at 7 days post-injury (dpi) significantly reduced collagen accumulation and fibrotic scarring induced by BLM in mice (Figure 1B). Histologic analysis of mouse lungs by H&E staining showed the gross destruction of normal lung tissue morphology due to chronic injury and pathologic scarring at 28 dpi in the BLM group. However, when compared with the BLM alone group, lung tissues from BLM + rFGF10 mice had much less architectural destruction and fibrosis (Figure 1B). We further analyzed the H&E sections to compare the extent of fibrosis in BLM + rFGF10 versus BLM lungs according to Ashcroft’s method [31]. As anticipated, rFGF10 treatment reduced the Ashcroft’s fibrosis score in response to BLM treatment (Figure 1C,D). Treatment with rFGF10 alone had no apparent effects on normal mouse lung tissue, as visualized by H&E and Masson staining (Figure S3). To further evaluate the extent of fibrosis, Masson’s trichrome and IHC staining were performed to determine collagen deposition and α-smooth muscle actin (α-SMA) expression in mouse lungs. In addition, hydroxyproline, the major constituent of collagen, was also measured. As shown in Figure 1B,E,F, i.t. instillation of BLM led to a significant increase in collagen deposition and α-SMA expression, whereas rFGF10 administration significantly attenuated the BLM-induced damage. Consistent with these findings, the hydroxyproline content was also found to be markedly increased in BLM-alone mice, but was significantly lowered in BLM + rFGF10 mice, suggesting that treatment with rFGF10 inhibited the BLM-induced hydroxyproline accumulation (Figure S2). Furthermore, coadministration of rFGF10 at day zero also significantly reduced collagen accumulation and fibrotic scarring induced by BLM in mice (Figure S4), suggesting that i.t administration of rFGF10 at an early phase of BLM injury attenuates fibrosis formation. 

To verify whether the effects of rFGF10 were mediated via FGFR2b activation, we used the [*Sftpc^CreERT2^/^+^*; *tdTomato^flox/+^*] mice to examine AT2-lineage labeled cells across different groups. In our experimental approach, mice received three i.p. injections of 200 μg tamoxifen/g (body weight) every other day to induce CreERT2 translocation to the nucleus and subsequent Cre-mediated recombination of the *Lox-STOP-Lox-tdTomato* allele located in the *Rosa26* locus, thereby leading to constitutive tdTomato expression in all Sftpc^Pos^ cells. After one week of washout following tamoxifen induction, BLM injury was performed. Mice were sacrificed at 7 dpi (Sham and BLM group), 7 dpi + 12 h (BLM + rFGF10 group), and 28 dpi (BLM group). A quantitative analysis of pFGFR2b^Pos^ tdTom^Pos^ DAPI^Pos^/tdTom^Pos^DAPI^Pos^ by immunofluorescence staining was carried out (Figure 1G,H). Note that it is not technically possible to distinguish between IAAPs and AT2s based on tdTomato expression by immunofluorescence [22]. Our results indicated that in the non-injured lung, around 30% of the lineage-traced cells are positive for pFGFR2b, indicating that FGFR2b signaling is active during homeostasis. Following BLM administration, this percentage fell to 15% and 10% at 7 and 28 dpi, respectively, suggesting the loss of FGFR2b signaling. Interestingly, rFGF10 administration at 7 dpi maintained FGFR2b signaling at close to normal levels in lineage-labeled cells 12 h later. Taken together, these results suggest that the administration of rFGF10 during the early phase of BLM-induced injury (0–7 days) prevents the decrease in lineage-labeled cells undergoing FGFR2b signaling and is associated with decreased fibrosis formation.

### 3.2. Therapeutic rFGF10 Delivery at 21 dpi Accelerates Fibrosis Resolution

Fibrosis formation is usually observed by day 14 following BLM exposure in the mice, with the maximal pathological responses around 14–21 dpi. Therefore, to explore the therapeutic potential of exogenous rFGF10 on BLM-induced pulmonary injury following fibrosis formation, mice were administered rFGF10 at 21 dpi and sacrificed at 28 dpi, as depicted in Figure S5A. H&E staining of lung sections revealed that exogenous rFGF10 treatment significantly attenuated the BLM-induced pathological changes, such as the distortion of lung morphology and fibrotic scarring. This result was further supported by an improved Ashcroft score, indicating the alleviated severity of fibrosis (Figure S5B,C) and the reduced area of fibrotic lesions in the BLM + rFGF10 group versus the BLM-only group (Figure S5B,D). To further evaluate the effects of rFGF10 in pulmonary fibrosis, we next determined the expression of collagen and α-SMA in different groups by Masson’s trichrome, IHC, and WB. As expected, collagen and α-SMA expression levels were increased in the BLM injury group. In contrast, treatment with rFGF10 markedly reduced the BLM-induced increases in fibrotic markers α-SMA and collagen in vivo (Figure S5B,E–G). These data suggest a therapeutic potential of exogenous rFGF10 in the treatment of IPF.

### 3.3. rFGF10 Promotes Alveolar Epithelial Progenitor Cell Proliferation and Alveolar Repair

**Figure 2:**
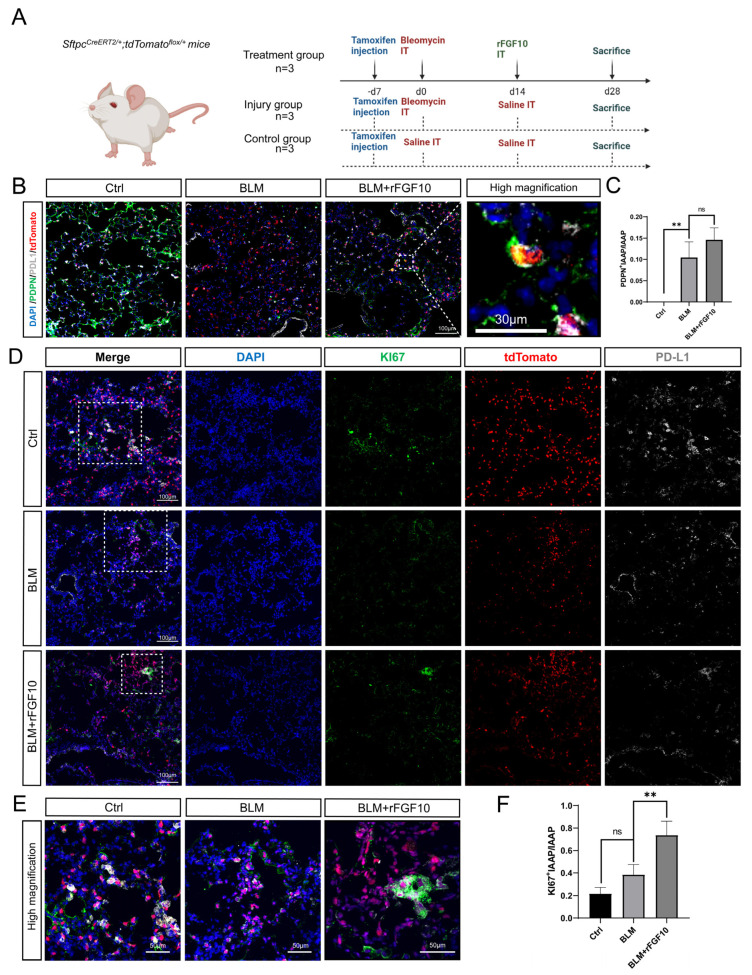
rFGF10 promotes alveolar epithelial progenitor cell proliferation and alveolar repair. (**A**) Timeline of tamoxifen, BLM and rFGF10 treatment of Sftpc^Pos^ lineage-labeled mice. BLM was administered in 2-month-old *Sftpc^CreERT2/+^*; *tdTomato^flox^^/+^* mice (*n* = 3). rFGF10 was administered 14 days after BLM administration in the treatment group (*n* = 3). Control mice were administered saline (*n* = 3). These mice were sacrificed at 28 dpi of BLM or saline administration (designated as Day 0). (**B**) Immunostaining of control, BLM, and BLM + rFGF10 groups of mouse lungs for PDPN (green) and PD-L1 (grey) at 28 dpi of BLM injury. (**C**) Quantification and ratio of PDPN^+^IAAPs in total IAAP cells. (**D**) Immunostaining of lung sections from mice for KI67 (green) and PD-L1 (grey) at 28 dpi. (**E**) Magnification of figure D. (**F**) Quantification and ratio of KI67^+^IAAPs in total IAAP cells. **: *p* < 0.01; ns: no significance.

Using the *Sftpc^CreERT2^*; *tdTomato* lineage-traced mice, we recently reported a novel AT2-IAAP subpopulation characterized by low *Sftpc* expression but that is enriched for PD-L1 expression [22]. IAAP cells, which represent quiescent and immature epithelial progenitors, undergo activation and expansion following PNX injury [22]. We recently reported the deletion of *Fgfr2b* in Sftpc^Pos^ cells and demonstrated that FGFR2b signaling is necessary for the survival of mature AT2s (tdTom^High^). Interestingly, IAAPs were concomitantly activated and proliferated [28]. Furthermore, IAAPs displayed upregulated *Fgfr2b* and *Etv5* expression, suggesting that these cells not only escaped *Fgfr2b* deletion by a mechanism that remains to be identified but responded to FGFR2b signaling. To explore whether IAAPs can also be activated by rFGF10 to play a progenitor role after BLM injury, *Sftpc^CreERT2/+^; tdTomato^flox/+^* mice were used to label the AT2 lineage, rFGF10 was administered at 14 dpi, and all mice were sacrificed at 28 dpi (Figure 2A). Co-staining for the canonical AT1 marker PDPN and PD-L1 showed that labeled AT2s distributed sporadically throughout the alveolar region in the control group, as shown in Figure 2B. BLM administration caused gross damage to both AT1 (PDPN) and AT2 (SFTPC) cells relative to the control group. However, rFGF10 administration showed a reparative re-organization effect on both cell types and alveolar structures. Furthermore, we identified tdTomato^Pos^ cells that co-stained for PDL1 and PDPN in BLM + rFGF10 mice, suggestive of a PD-L1-positive precursor to these cells, presumably IAAPs (Figure 2B). Figure 2C shows the ratio of PDPN^+^IAAPs in total IAAPs in the BLM group, which remained statistically higher, although in the BLM + rFGF10 group compared to the BLM group, no statistical significance was observed. In addition, co-staining of lung sections for tdTomato, PD-L1, and the proliferation marker Ki67 revealed an increased number of ki67-enriched clusters of PD-L1^Pos^ tdTomato^Pos^ cells in BLM + rFGF10 mice when compared to BLM-alone and control mice, suggesting that the clustering may have been a result of a proliferative response. In contrast, under the action of rFGF10, cells co-expressing SFTPC, PD-L1 and Ki67 were statistically enriched in areas of alveolar damage (Figure 2D–F). Altogether, these results suggest that in the context of BLM injury, rFGF10 may promote the proliferation of IAAPs, which will later differentiate into AT1s. 

### 3.4. Dynamic Alteration of IAAP Population during BLM-Induced Lung Fibrosis and Resolution

**Figure 3:**
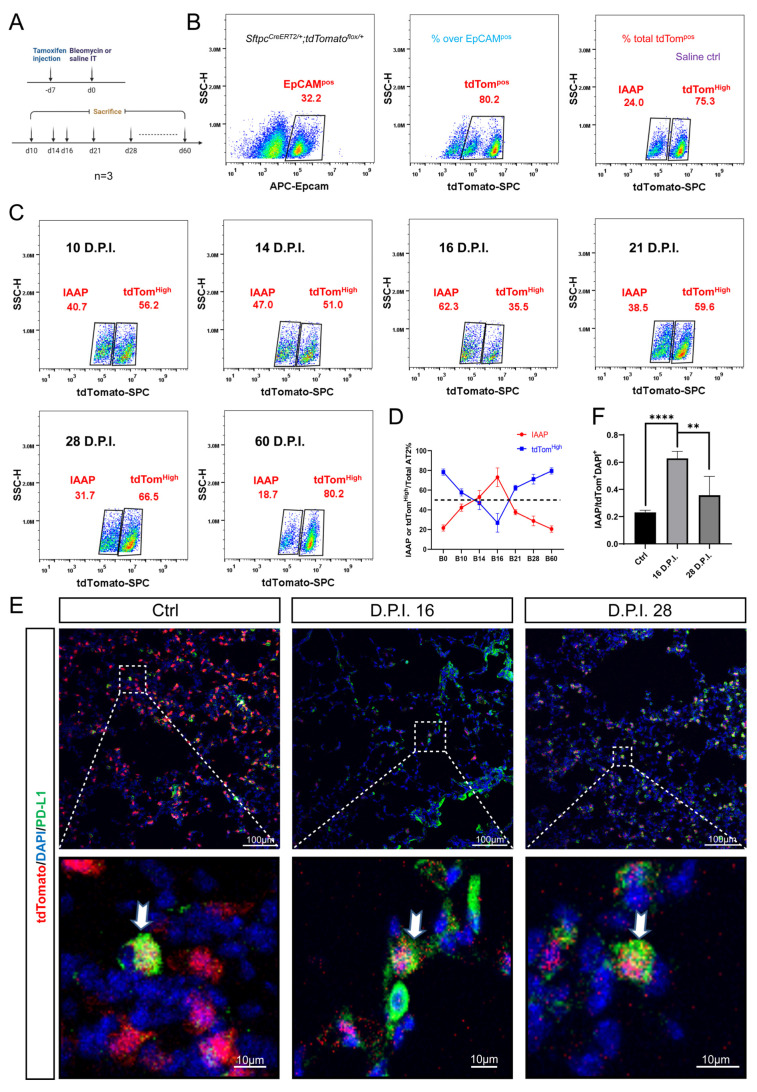
IAAPs are activated in response to BLM. (**A**) Timeline of tamoxifen treatment *of Sftpc^CreERT2^*^/+^, *tdTomato^flox/+^* mice (*n* = 4). BLM was i.t. administered in 2-month-old *Sftpc^CreERT2/+^*, *tdTomato^flox/+^* mice. Control mice were administered saline. For analysis, these mice were sacrificed 28 days after BLM (BLM) or saline administration (designated as Day 0). (**B**) Representative flow cytometry of EpCAM-positive population selection and the identification of IAAPs and mature AT2s in the control group. Flow chart shows the percentage of IAAPs and AT2s in total tdTomato^Pos^ cells. (**C**) Representative flow cytometry analysis of IAAPs and AT2s populations at 10, 14, 16, 21, 28 60 days after BLM administration. (**D**) Dynamic ratio change based on the quantification of IAAPs and AT2s percentages in total tdTomato^Pos^ cells at different times after BLM-induced injury (*n* = 3). (**E**) Immunostaining for PD-L1 on *Sftpc^CreERT2/+^*; *tdTomato^flox/+^* mouse lungs that received i.t. BLM at 2 months of age and were harvested at 16 and 28 dpi. (**F**) Quantification of the immunofluorescence showing a ratio of IAAPs in AT2 cells at 16 and 28 dpi compared to the control group. Data are presented as mean ± SEM. **: *p* < 0.01; ****: *p* < 0.0001.

Next, we sought to determine whether the mature AT2s and IAAPs are differentially involved in BLM-induced injury using the *Sftpc^CreERT2/+^; tdTomato^flox/+^* mice to label the AT2 lineage (Figure 3A). The lungs of euthanized mice were processed for single-cell suspensions at 14, 21, and 28 dpi. After removing CD31^Pos^ and CD45^Pos^ cells by MACS to enrich for the epithelial population, the cells were stained with APC-EpCAM antibodies and subjected to FACS analysis. We showed that the proportion of EpCAM^Pos^ cells as a percentage of total cells was 32.2%, of which the percentage of tdTom^Pos^ cells was 80.2% in the control mice. IAAPs and mature AT2s represented 24.0% and 75.3%, respectively, of the overall tdTom^Pos^ cells in the control mice (Figure 3B). Interestingly, the relative ratio of IAAPs to tdTom^Pos^ AT2s was increased to 47.0% in the 14 dpi group, and this ratio decreased to 38.5% at 21 dpi, then 31.7% at 28 dpi, which is still higher than that of the control group. In contrast, the tdTom^High^ AT2 population decreased to 51.0% at 14 dpi, rebounded to 59.6% at 21 dpi, and continued to rise at 28 dpi (66.5%) (Figure 3C). Our data suggest that, when compared to the tdTom^High^ AT2s, IAAPs are more resistant to BLM injury and are rapidly activated to proliferate. To determine whether this ratio peaked at 14 dpi and whether it was likely to return to normal ratios over time, we further sacrificed the post-BLM mice at 10, 16, and 60 dpi (Figure 3C). FACS results showed the ratio of IAAPs was also increased at 10 dpi (47.0%), peaked at 16 dpi (62.3%) and returned to normal levels at 60 dpi (18.7%) as shown in Figure 3C,D. Consistent with our flow cytometry data, immunofluorescence staining for PD-L1 revealed more IAAPs at 16 dpi in the lungs of BLM-treated mice when compared to the control and 28 dpi groups (Figure 3E,F). Altogether, our data reveal that tdTom^High^ AT2 cells represent the majority of total AT2s during homeostasis. However, the situation changes in response to BLM, whereby IAAPs become activated and the IAAP/tdTom^High^ ratio dynamically changes.

### 3.5. rFGF10 Administration Triggers Further IAAPs Expansion

**Figure 4:**
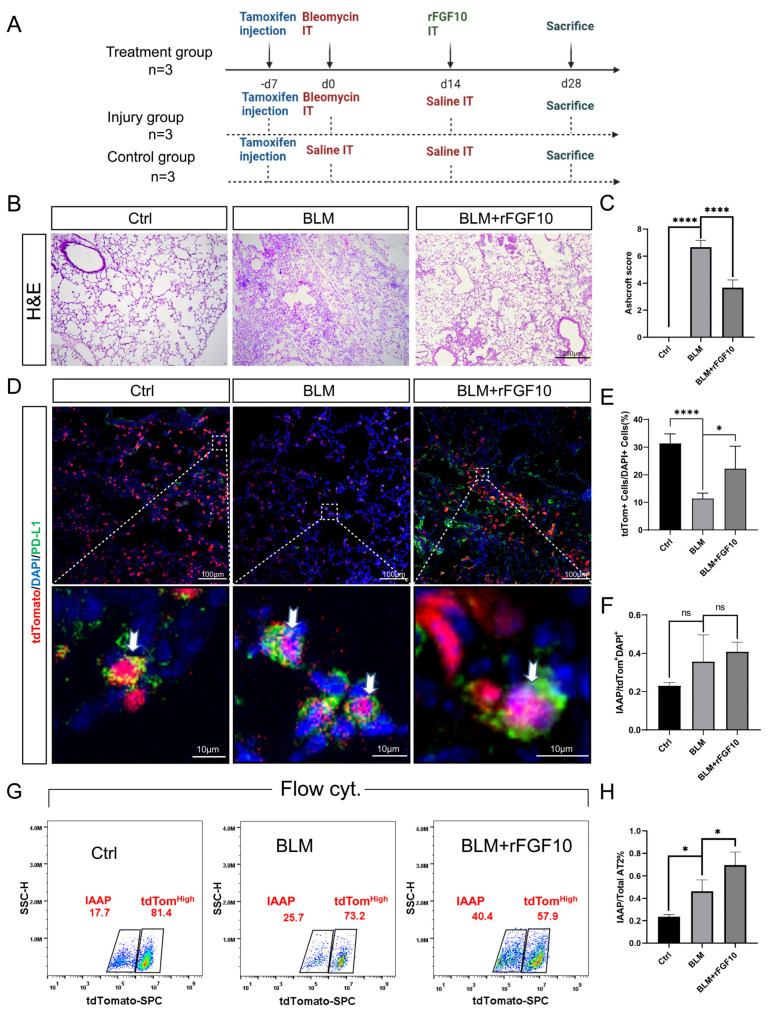
Further expansion of IAAPs following rFGF10 administration. (**A**) Timeline of tamoxifen treatment of *Sftpc* lineage traced mice (*n* = 3). BLM was administered in 2-month-old Sftpc^CreERT2/+^, *tdTomato ^flox/+^* mice (BLM). rFGF10 was administered at 14 dpi in post-BLM mice (BLM + rFGF10). Control mice were administered saline (Ctrl). For analysis, these mice were sacrificed at 28 dpi. (**B**) H&E staining of lung sections (original magnification, ×20) of ctrl, BLM, BLM + rFGF10 group. (**C**) Semi-quantitative analyses of lung tissue using Ashcroft score (*n* = 3). Note the score was markedly decreased in the BLM + rFGF10 group (*p* < 0.0001). (**D**) Immunostaining for PD-L1 on control, BLM, and BLM + rFGF10 of *Sftpc^CreERT2/+^*; *tdTomato^flox/+^* lungs. (**E**) Quantification and ratio of tdTomato^+^ cells in total cells. (**F**) Quantification of the immunofluorescence showing the expression of PD-L1 in lineage-labeled cells for the indicated mouse groups. (**G**) Representative FACS analysis of IAAP and tdTom^High^-AT2 populations of ctrl, BLM and BLM + rFGF10 at 28 dpi. (**H**) The quantification of IAAPs/tdTom^High^-AT2s ratio of all tdTom^pos^ cells. Data are presented as mean ± SEM. *: *p* < 0.05; ****: *p* < 0.0001.

Given the potent effect of rFGF10 in promoting alveolar epithelial progenitor cell proliferation and alveolar repair, whether IAAPs are further activated in the presence of rFGF10 is a critical question. Post-BLM mice were administered rFGF10 at 14 dpi and sacrificed 2 weeks later at 28 dpi. The entire lungs were then sectioned for immunostaining or processed for cell isolation and FACS analysis. Our treatment scheme is depicted in Figure 4A. Consistent with the beneficial effect of rFGF10 shown in Figure 1B and Figure 2B, rFGF10 given at 14 dpi similarly attenuated pulmonary fibrosis as indicated by the significantly lower Ashcroft score (Figure 4B,C). Also, our immunofluorescence staining data revealed marginally increased IAAPs in BLM-treated mice when compared to the saline control, as well as the BLM + rFGF10 group compared to the BLM group, albeit no statistical significance was observed (Figure 4D,F). The ratio of tdTomato^+^ cells in total cells shows that BLM administration caused gross damage to AT2 (SFTPC) cells relative to the control group and rFGF10 administration showed a reparative re-organization effect in the AT2 cells (Figure 4E). Flow results showed that IAAPs and tdTom^High^-AT2s represented 17.7% and 81.4%, respectively, of the overall AT2 cells in the control group, whereas in the BLM group, IAAPs and tdTom^High^-AT2s represented 25.7% and 73.2%, respectively, of the overall tdTom^Pos^-AT2s, reflecting a significant increase in the IAAP subpopulation with a concomitant decrease of tdTom^High^-AT2s. Additionally, in the BLM + rFGF10 group, out of total AT2s, 40.4% were IAAPs and 57.9% were tdTom^High^-AT2s (Figure 4G) which represents a further and persistent increase in the ratio of IAAPs over tdTom^High^-AT2s in the post-BLM mice by rFGF10 treatment (Figure 4H). When combined with the histological and immunofluorescence results, we hypothesized that IAAPs, rather than tdTom^High^-AT2s, are the significant contributors during lung repair, and rFGF10 has a significant and sustained impact on the relative and dynamic change between the two subpopulations. 

### 3.6. rFGF10 Increases the Duration of IAAP/AT2 Population Ratio

**Figure 5:**
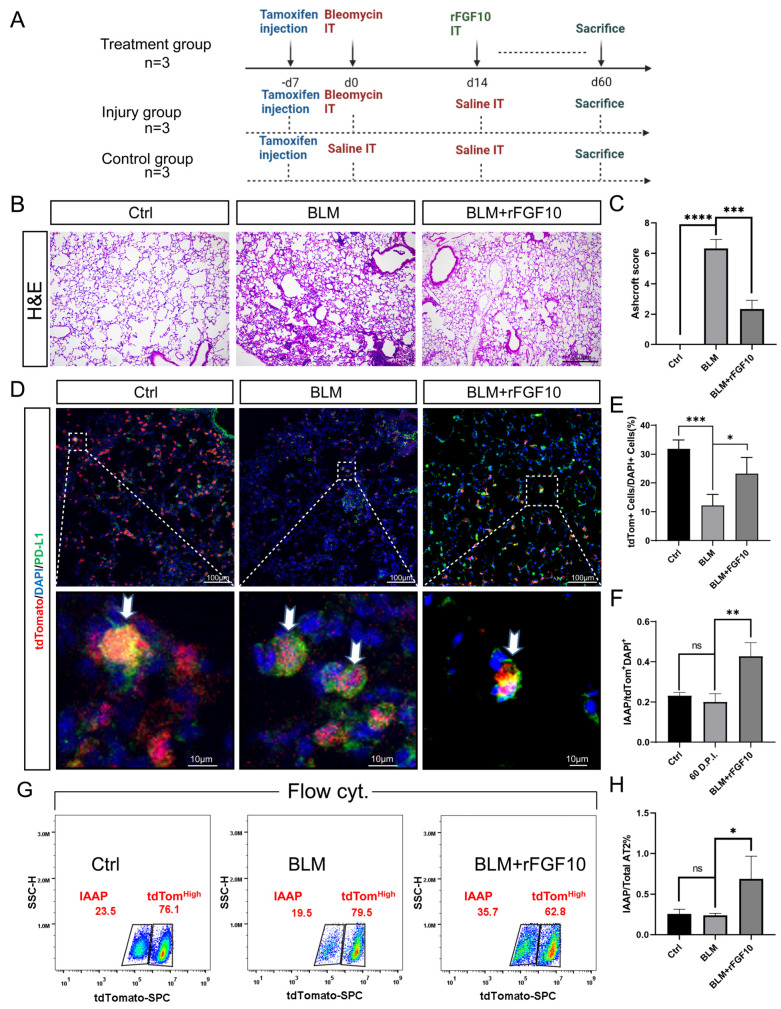
rFGF10 leads to a persistent increase in the IAAPs/AT2s ratio. (**A**) Timeline and treatment scheme of *Sftpc^Pos^* lineage-labeled mice (*n* = 3). BLM was administered in 2-month-old *Sftpc^CreERT2/+^, tdTomato^flox/flox^* mice. For the BLM + rFGF10 group, rFGF10 was administered in post-BLM mice at 14 dpi. For analysis, these mice were sacrificed at 60 dpi after BLM or saline administration (designated as Day 0). (**B**) H&E staining of 5-µm-thick lung sections (original magnification, ×20) of ctrl, BLM, BLM + rFGF10 group. (**C**) Semi-quantitative analyses of lung tissue using Ashcroft score (*n* = 3). Note the markedly decreased score in the BLM + rFGF10 group (*p* < 0.001). (**D**) Immunostaining for PD-L1 on control, BLM, and BLM + rFGF10 of *Sftpc^CreERT2/+^*; *tdTomato^flox/+^* lungs. (**E**) Quantification and ratio of tdTomato^+^ cells in total cells. (**F**) Quantification of the immunofluorescence showing the expression of PD-L1 in *Sftpc^Pos^* lineage-labeled cells for the indicated mouse groups. (**G**) Representative FACS analysis results of IAAPs and tdTom^High^-AT2s subpopulations at 60 dpi of control and post-BLM administration. (**H**) Quantification and ratio of IAAPs^/^tdTomato^H^^igh^-AT2s ratio in total tdTom^Pos^ cells. Data are presented as mean ± SEM. *: *p* < 0.05; **: *p* < 0.01; ***: *p* < 0.001; ****: *p* < 0.0001.

The results shown in Figure 4 indicated that rFGF10 administration enhanced the magnitude of the IAAPs to tdTom^High^-AT2 cell ratio when compared to the BLM-alone group, which was not restored to the control level at 28 dpi. We, therefore, further extended our observation of the potential impact of rFGF10 administration on IAAP and mature AT2(tdTom^High^) dynamic change to 60 dpi. Our experimental scheme is depicted in Figure 5A. Histological analysis revealed apparent remaining lung scarring at 60 dpi in the BLM-alone group. Although these scars were incompletely resolved in the BLM + rFGF10 lungs, the remaining fibrosis was less severe, as evidenced by the significantly lower Ashcroft score than the BLM-alone group (Figure 5B,C). Furthermore, immunofluorescence analysis showed that rFGF10 administration at 14 dpi greatly enhanced the duration and the magnitude of the IAAPs to overall tdTom^Pos^-AT2s ratio when compared to the BLM-alone control at 60 dpi in the region of injury (Figure 5D–F). Notably, although the relative proportion of IAAPs over tdTom^Pos^-AT2 returned to normal levels in the BLM-alone group (19.5%), such ratios in the BLM + rFGF10 group were 35.7% (Figure 5G), which remained statistically higher than the BLM-alone group suggesting a persistent IAAPs activation (Figure 5H). Altogether, these results indicate that rFGF10 may exert beneficial effects in the context of BLM injury via enhancing IAAPs’ proliferation and mediated regeneration.

## 4. Discussion

Herein, we demonstrated that, depending on the timing of administration, exogenous rFGF10 exhibited robust preventative or significant therapeutical efficacy toward BLM-induced fibrosis. Therefore, our findings provide fundamental preclinical data for the potential clinical uses of rFGF10 in IPF. While this work is ongoing, another line of our research revealed an essential role of FGFR2b signaling in maintaining the AT2 lineage during adult lung homeostasis. In this model of AT2 injury, we observed a drastic drop in mature AT2s, which underwent apoptosis upon the targeted deletion of *Fgfr2b* in the *Sftpc^Pos^* lineage [28]. On the contrary, IAAPs not only managed to survive and expand (despite being also targeted for *Fgfr2b* deletion) but also displayed enhanced alveolosphere formation in vitro [28]. How IAAPs responded to FGFR2b signaling and escaped *Fgfr2b* deletion remains to be further investigated. Our most recent study further identified the human equivalent of the IAAPs, which were amplified in IPF patients [27]. These findings are consistent with our initial description of this newly identified AT2 subpopulation as becoming activated and proliferative in the context of PNX-induced injury [22]. Overall, our results using rFGF10 as a therapeutic approach are highly consistent with the previously reported beneficial properties of endogenous FGF10/FGFR2b signaling in promoting epithelial repair and regeneration from various injuries such as naphthalene, hyperoxia in neonates (to mimic a bronchoalveolar dysplasia-like phenotype) and BLM-induced fibrosis [25]. The beneficial effects of rFGF10 likely reflect the net outcome of its impact on different epithelial cell types and their interplay with the surrounding mesenchymal cells, as exemplified by AT2/lipofibroblast interactions that we recently reviewed [32].

As a newly identified AT2 subpopulation, the regulation of IAAPs remains to be further characterized molecularly and functionally. Given the critical importance of AT2s in injury repair and fibrosis and the progenitor-like properties of IAAPs, we tested the hypothesis that IAAPs would be responsive and dynamically regulated in the course of BLM injury. We first attempted to determine whether IAAPs can be mobilized after BLM-induced injury and, for the first time, established the dynamic profile of IAAPs versus the canonical mature AT2s in the course of BLM-induced fibrosis development and resolution. Indeed, contrary to the drastic loss of mature AT2s following BLM injury, the relative proportions of the IAAPs were markedly amplified, with the ratio of IAAPs/EpCAM peaking at 16 dpi, reflecting a six-fold increase in the ratio of IAAPs/tdTom^High^-AT2s when compared to the non-injured controls. BLM injury elicited an initial rise in the IAAPs/EpCAM ratio, which was apparent at 10 dpi, peaking at 16 dpi before slowly returning to baseline by 2 months. In contrast, tdTom^High^-AT2 exhibited the opposite trend concurrently, reflecting the loss of mature AT2s. Significantly, although tdTom^High^-AT2 cells express higher levels of *Fgfr2b*, rFGF10 administration preferentially enhanced the duration and extent of the IAAPs/EpCAM ratio. Together with previous studies, the current data suggest that IAAPs may be the primary source of regenerative activity in the alveolar region, rather than the previously thought mature bona fide Sftpc^High^-AT2s. Further studies will be required to validate the relative importance of these two distinct AT2 subpopulations. Although our findings reveal a potential therapeutic role for exogenous rFGF10 in IPF, as well as new insight about IAAP/mature AT2 ratio dynamic alterations in response to BLM injury, whether the beneficial effects of rFGF10 on BLM-induced fibrosis are indeed mediated through the enhancement of IAAP proliferation is not entirely conclusive. It is vital to generate novel lineage-tracing strategies that allow for the specific labeling of *Sftpc/Pd-l1* double-positive cells to address these questions. In this regard, a dual recombinase-mediated genetic lineage tracing system to specifically track *Pd-l1^Pos^ Sftpc^Pos^* AT2 subpopulations will be needed to gain deeper insights into the various biological aspects of IAAPs in the normal and diseased lung. 

A puzzling question related to the behavior of IAAPs in end-stage IPF is their lack of contribution to the mature AT2 lineage. These cells are indeed amplified in IPF but seem to be stalled in their differentiation status [27]. One possibility explaining this failure to differentiate could be linked to the high inflammatory status of these lungs. Inflammation has been shown to prevent the differentiation of KRT8^Pos^ intermediate AT2s into AT1 in mice [33]. Similar intermediate populations of human AT2 have recently been reported upon co-culture with human mesenchymal cells [34]. These cells are defined by the differential expression of a set of epithelial markers including SFTPC, KRT5, KRT8, KRT17, as well as TP63, in response to fibrotic signaling. Such a mechanism is proposed to underlie the functional AT2 loss and expansion of alveolar metaplastic KRT5^Pos^ basal cells, which are highly pertinent to human IPF pathogenesis. The potential relationship of these human AT2 intermediate populations with IAAPs in response to lung injuries and whether the same process is at work in IAAPs remains to be illustrated in the future. Exogenous rFGF10 treatment, which delivered a significant therapeutic efficacy toward BLM-induced fibrosis, likely accelerated the restoration of AT2 homeostasis, whereas the mobilization of IAAPs, as indicated by both increased peak ratio and the duration of IAAP expansion relative to EpCAM^Pos^ cells, may contribute to such a process. Additionally, upon in vitro co-culture with Sca1^Pos^ residential mesenchyme, these IAAP cells displayed enhanced alveolosphere formation and increased their AT2 signature drastically, suggesting their differentiation towards mature AT2s [28]. 

Another puzzling question is whether the IAAPs have a direct impact on fibroblast behaviors and expansion. The precise nature of the interaction between alveolar epithelial stem cells (AT2s or IAAPs) and relevant mesenchymal cells during homeostasis is still largely unknown. In addition, how alveolar epithelial cells impact mesenchymal niches that have undergone a transition towards the activated myofibroblast phenotype present in fibrotic foci, to trigger their differentiation towards a benign phenotype, is largely unknown. In this context, co-culturing IAAPs with lung fibroblasts could be considered. However, a first difficulty to carry out this experiment deals with the IAAPs themselves. We have previously isolated IAAPs from non-injured lungs [22] and from lungs displaying deletion of *Fgfr2b* in AT2s [27]. The deletion of Fgfr2b in AT2s leads to a situation similar to what we showed in the context of bleomycin treatment: we observed a significant drop in Tom^High^ (mature) AT2s and an increase in IAAPs. An in vitro culture of the IAAPs from the non-injured lung with Sca1^Pos^-resident mesenchymal cells (rMC, CD31/CD45/EpCAM triple negative) from wild-type lungs shows that IAAPs display very low level of proliferative capability, therefore confirming the results obtained in vivo that these cells are quiescent. On the other hand, an in vitro culture of the IAAPs from lungs displaying deletion of *Fgfr2b* in AT2s with resident Sca1^Pos^ mesenchymal cells from wild-type lungs shows that injured IAAPs display an enhanced proliferative capability, albeit at a much lower level of what was expected to compensate for the loss of AT2s. This leads to the question as to whether the 3D model used (IAAPs with Sca1^Pos^ rMC) is sufficient to capture the full behavior of the IAAPs. Among the missing components potentially allowing a full response are immune cells, as well as the damage associated molecular patterns (DAMPs) released by dying AT2 cells. Without these components, any attempt to carry out in vitro co-culture experiments between IAAPs and lung fibroblasts is bound to be disappointing.

Another challenge linked to the in vitro co-culture experiment deals with the nature of the fibroblasts to be used. The fibroblasts still constitute a big black box and are made of many subtypes. The alveolar fibroblasts, and in particular the Fgf10^Pos^ Sca1^Pos^ rMC, which likely represent the so-called lipofibroblasts, are likely the ones undergoing a transition towards the activated myofibroblast phenotype present in fibrotic conditions [35,36]. Ideally, this is the mesenchymal cell subtype that needs to be isolated from bleomycin lungs to co-culture them with the IAAPs. A major problem is that activated myofibroblasts do not support the proliferation of non-injured AT2s (or IAAPs for that matter), limiting the use of this in vitro model. Therefore new in vitro models need to be established. Such models include, for example, human embryonic lung cell lines as well as primary cultures of human lung fibroblasts from either donor or IPF lungs to study the impact of signals inducing their differentiation towards the alveolar fibroblasts/lipofibroblast phenotype, such as with metformin [37] or towards the activated myofibroblasts phenotype (for example, upon treatment with TGF-β1). Once the cellular, transcriptional and metabolic readouts have been clearly delineated, co-culture experiments with either AT2s or IAAPs can be carried out. 

Interestingly, the fact that the IAAPs responded to rFGF10, indicated by the increase in their *Fgfr2b* expression following injury, can be translated into increased FGFR2b signaling. Therefore, FGFR2b signaling likely contributes to compensate for the lost mature AT2s caused by BLM injury. However, further studies using, for instance, IAAP-specific lineage-tracing and targeted cell ablation, are needed to determine the causal role of IAAPs in lung injury/repair, and whether FGF10 is a causal factor in IAAP expansion after injury. Elucidating the molecular mechanisms underlying IAAPs’ activation, proliferation, and differentiation into mature AT2s, and their sequential capacity for further differentiation into AT1 cells, will be critical to promote optimal injury repair. Overall, the present study suggests that the exogenous administration of rFGF10 as well as harnessing the reparative capacity of IAAPs holds potential as a future therapeutic strategy in the treatment of IPF.

## Data Availability

The data presented in this study are available on request from the corresponding author.

## Supplementary Materials

The following supporting information can be downloaded at: https://www.mdpi.com/article/10.3390/cells11152396/s1, Figure S1: FGF10 is overexpressed in bleomycin injury group; Figure S2: rFGF10 reduces hydroxyproline content; Figure S3: rFGF10 have no effect on normal lung tissue; Figure S4: rFGF10 exhibits preventive efficacy toward BLM-induced injury; Figure S5: rFGF10 exhibits therapeutic efficacy toward BLM-induced fibrosis.
