# Supplementary material for: FGF10 Therapeutic Administration Promotes Mobilization of Injury-Activated Alveolar Progenitors in a Mouse Fibrosis Model
Source: Cells. 2022 Aug 3;11(15):2396. doi: 10.3390/cells11152396 (PMC9368687; doi:10.3390/cells11152396)
Supplement: Supplementary file 1 [file cells-11-02396-s001.zip › cells-1841368-supplementary.pdf]

cells

MDPI

Supplementary Figure S1

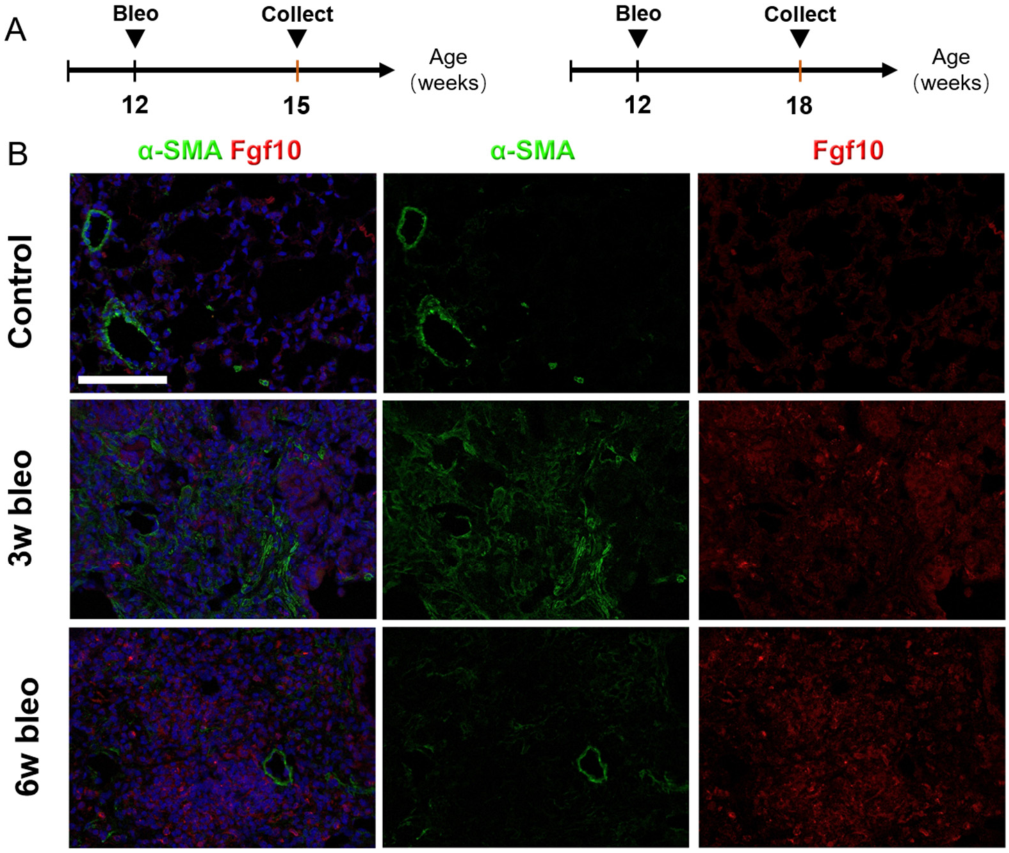

Supplementary Figure S1. FGF10 is overexpressed in bleomycin injury group. A. Experimental strategy. **B.** Immunostaining on lungs for FGF10 (red), α-SMA (green) at control，3 weeks and 6 weeks after bleomycin injury groups.

Supplementary Figure S2

A

Optical Density(550nm)

Blank	0.072	0.073	0.072
5μg/ml HYP	0.368	0.362	0.367
Ctrl	0.206	0.185	0.196
BLM+FGF10	0.231	0.259	0.191
BLM	0.282	0.21	0.222

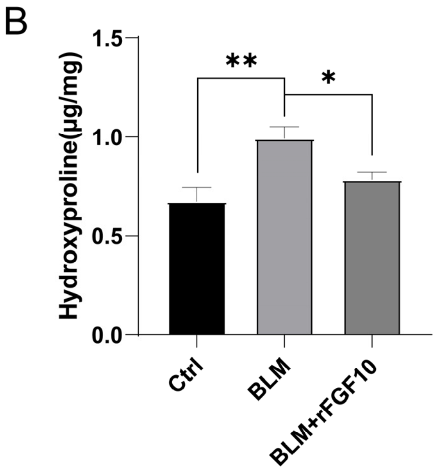

Supplementary Figure S2. rFGF10 reduces hydroxyproline content. Optical Density of Control，BLM+rFGF10 and BLM groups. B. Quantification of hydroxyproline content in the lung tissues of each mouse group (n = 3). Data are presented as mean ± SEM. ∗: p<0.05; ∗∗: p<0.01.

Supplementary Figure S3

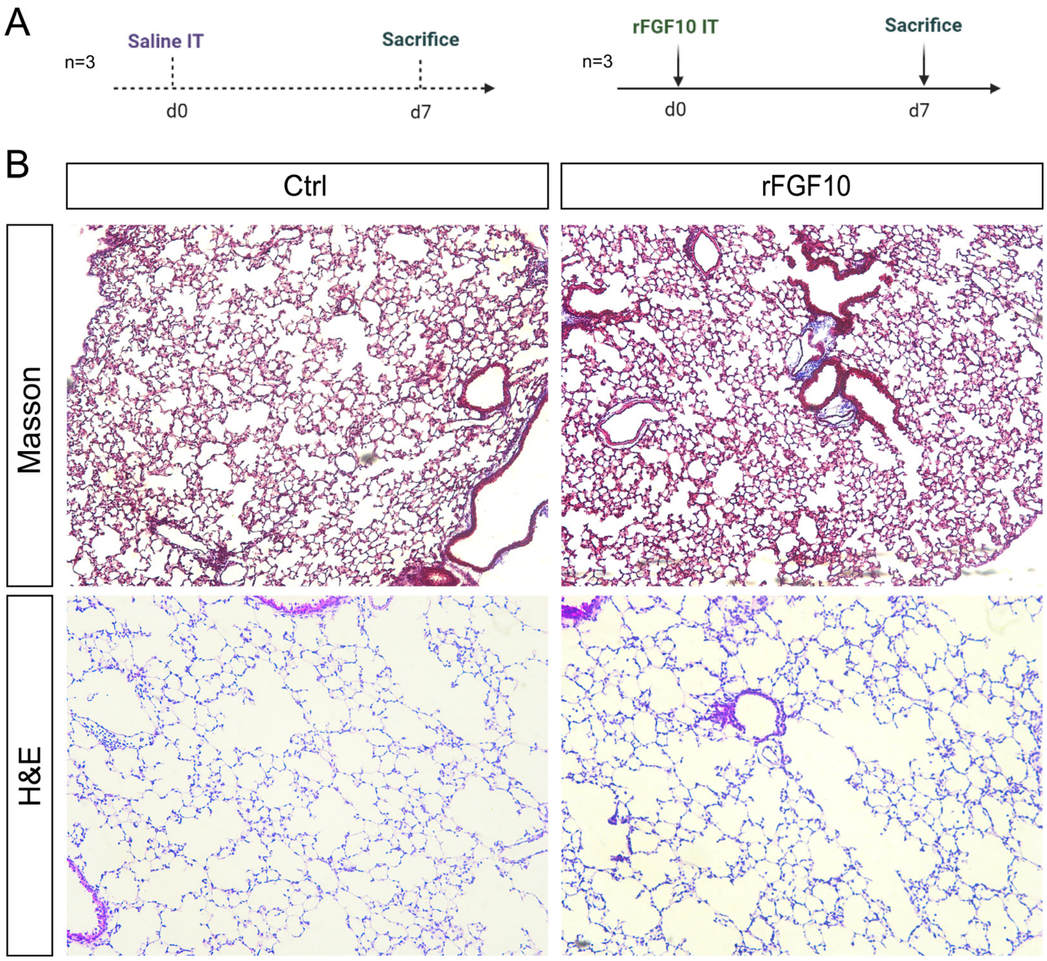

Supplementary Figure S3. rFGF10 have no effect on normal lung tissue. Timelines of rFGF10 administration with saline as control, all the mice were euthanized 7 days after administration (designated as Day 0). **B.** H&E and Masson's trichrome of lung tissue from control and rFGF10 group mice (n=3).

Supplementary Figure S4

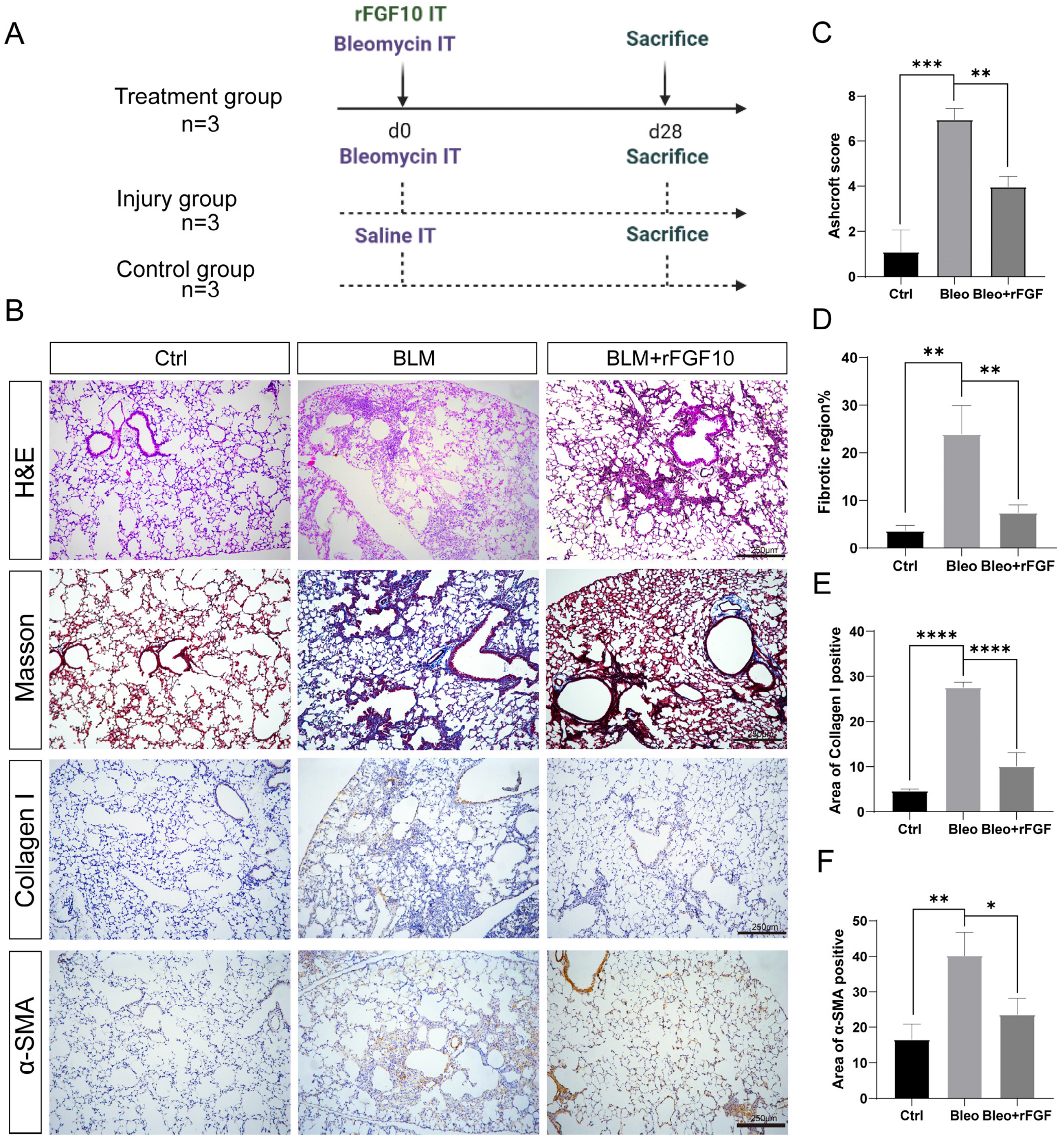

Supplementary Figure S4. rFGF10 exhibits preventive efficacy toward BLM-induced injury. A. Timelines of BLM and rFGF10 administration with saline as control, all the mice were euthanized 28 days after BLM administration (designated as Day 0). Histological analysis and quantitative fibrosis scoring of lung sections. **B.** H&E, Masson's trichrome and IHC staining (α-SMA, collagen) of lung tissue from control, BLM, BLM+rFGF10 group mice. **C.** Semi-quantitative analyses of lung tissue using Ashcroft score ($n = 3$). Note the highly significantly decreased score in the BLM + rFGF10 group compared to BLM alone. **D.** ImageJ quantification of fibrotic regions based on Masson's trichrome staining ($n = 3$). **E-F,** Quantification of collagen and α- SMA IHC staining in the lung sections of each mouse group ($n = 3$). Data are presented as mean ± SEM. ∗: $p<0.05$; ∗∗: $p<0.01$; ***: $p<0.001$; ****: $p<0.0001$.

Supplementary Figure S5

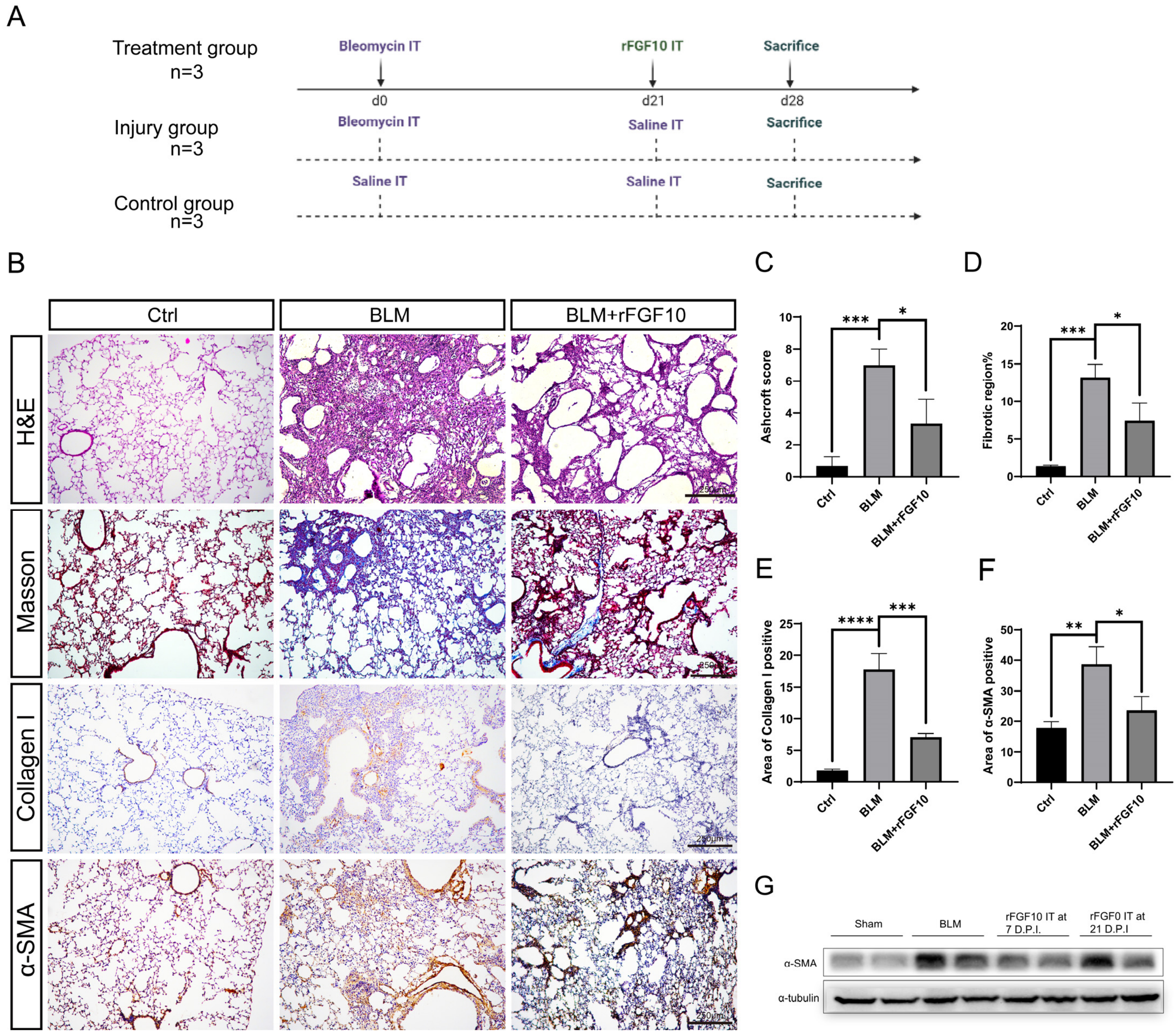

Supplementary Figure S5. rFGF10 exhibits therapeutic efficacy toward BLM-induced fibrosis. A. Experimental scheme for BLM and rFGF10 administration with saline as control. The mice were sacrificed at 28 dpi after BLM administration (designated as Day 0). Histological analysis and quantitative fibrosis scoring of lung sections. **B.** H&E, Masson's trichrome and IHC staining (α-SMA, collagen) of lung tissue from control, BLM, BLM+rFGF10 group mice. C. Semi-quantitative analyses of lung tissue using Ashcroft score (n = 3). The score was significantly decreased in the BLM + rFGF10 group. D. ImageJ quantification of fibrotic regions based on Masson's trichrome staining (n = 3). E-F. Quantification of collagen and α- SMA IHC staining in the lung sections of each mouse group (n = 3). G. Protein expression levels of collagen 1 and α- SMA in the lung tissue of mice subjected to BLM injury or treated with rFGF10 12 h at 7 dpi and 21 dpi, respectively, before harvesting. Data are presented as mean ± SEM. *: p<0.05; **: p<0.01; ***: p<0.001; ****: p<0.0001.
